# Feasibility and Reliability of SmartWatch to Obtain 3-Lead Electrocardiogram Recordings

**DOI:** 10.3390/s20185074

**Published:** 2020-09-07

**Authors:** Amirali Behzadi, Alireza Sepehri Shamloo, Konstantinos Mouratis, Gerhard Hindricks, Arash Arya, Andreas Bollmann

**Affiliations:** 1 Department of Electrophysiology, Heart Center Leipzig at University of Leipzig, 04289 Leipzig, Germany; alireza.sepehri-shamloo@helios-gesundheit.de (A.S.S.); Gerhard.Hindricks@helios-gesundheit.de (G.H.); Arash.Arya@helios-gesundheit.de (A.A.); Andreas.Bollmann@helios-gesundheit.de (A.B.); 2 Leipzig Heart Digital at Leipzig Heart Institute, 04289 Leipzig, Germany; Konstantinos.Mouratis@leipzig-heart.de

**Keywords:** smartwatch, Apple Watch, electrocardiogram, feasibility, reliability, wearables, mobile health

## Abstract

Some of the recently released smartwatch products feature a single-lead electrocardiogram (ECG) recording capability. The reliability of obtaining 3-lead ECG with smartwatches is yet to be confirmed in a large study. This study aimed to assess the feasibility and reliability of smartwatch to obtain 3-lead ECG recordings, the classical Einthoven ECG leads I-III compared to standard ECG. To record lead I, the watch was worn on the left wrist and the right index finger was placed on the digital crown for 30 s. For lead II, the watch was placed on the lower abdomen and the right index finger was placed on the digital crown for 30 s. For lead III, the same process was repeated with the left index finger. Spearman correlation and Bland-Altman tests were used for data analysis. A total of 300 smartwatch ECG tracings were successfully obtained. ECG waves’ characteristics of all three leads obtained from the smartwatch had a similar duration, amplitude, and polarity compared to standard ECG. The results of this study suggested that the examined smartwatch (Apple Watch Series 4) could obtain 3-lead ECG tracings, including Einthoven leads I, II, and III by placing the smartwatch on the described positions.

## 1. Introduction

The 12-lead electrocardiogram (ECG) is the most widely used tool for early diagnosis of heart diseases [1]. However, standard 12-lead ECG is a process that requires trained personnel and specialized equipment and cannot be performed without patients’ presence in health centers [2]. Additionally, as the elderly portion of the population will grow over the coming years, health centers are expected to experience a steady increase in demand for procedures like standard ECG, which can be challenging to handle.

Recent advances in remote health and wearable technologies have offered new opportunities for remote monitoring of patients [3,4,5]. One of the products of these advances is handheld/wearable single-lead ECG recorders [6]. The latest development in this area is the inclusion of single-lead ECG in smartwatches, which are getting more popular every day [7]. One of the smartwatch products with this capability is the Apple Watch Series 4, which can record single-lead ECG in 30 s using two positive and negative electrodes. The Apple Watch Series 4 was the first smartwatch with the capability of single-lead ECG recording that received a de novo Food and Drug Administration (FDA) clearance in August 2018 [8]. However, doubts about the quality of ECG signals generated by smartwatches and the fact that they are limited to one lead (similar to lead I) have largely limited their clinical application [9,10,11]. Considering the short time since the production of the Apple Watch Series 4, not many studies have been conducted regarding the quality assessment of the ECGs generated by this device. Our systematic search of the Pubmed database with the following search terms “Apple Watch”, “Smartwatch”, “Electrocardiogram”, and “ECG” in August 2020 result in 79 records. However, the feasibility of recording a 3-lead ECG with the Apple Watch Series 4 was reported in only one case report of two patients with myocardial infarction [7], a technical report [12], and three observational studies from a same group of investigators [13,14,15]. The other available studies were related to the other capabilities of smart wearables, ranging from screening and diagnostic to treatment functions, using other technologies, namely photoplethysmography (PPG) [4,5,16,17,18,19,20,21].

Although the technique of 3-lead ECG recording based on Einthoven’s triangle using the Apple Watch Series 4 has been described by these studies, no study has quantitatively evaluated the quality of recorded tracings by smartwatch compared to standard ECG, and our knowledge is limited to the qualitative comparison of the Apple Watch versus standard ECGS. Additionally, the possibility of generating 3-lead ECGs with smartwatches is yet to be confirmed in a large study with other study groups. In addition, a few studies have verified the ability of handheld ECG recorders to record different ECG leads [14,15], but the need for additional wires and adhesive ECG tabs have limited their use in practice.

Establishing the similarity of the 3-lead ECG signals generated by smartwatches to those recorded in standard ECG can greatly contribute to improve the diagnostic utility of smartwatch-generated ECGs, especially in cases where there is no immediate access to advanced diagnostic facilities. This study aimed to assess the feasibility and reliability of smartwatch to obtain 3-lead electrocardiogram recordings, the classical Einthoven ECG leads I-III compared to standard ECG.

## 2. Materials and Methods

### 2.1. Study Design and Setting

This was a sub-study of the Leipzig Apple Heart Rhythm Study conducted in 2019 on patients referring to the Leipzig Heart Center, Germany. The Leipzig Apple Heart Rhythm Study is a prospective, nonrandomized, adjudicator-blinded trial aiming to confirm the feasibility and reliability of ECGs generated by smartwatches for the diagnosis of cardiac arrhythmias, which has been registered on ClinicalTrials.gov (NCT04092985). Patients were asked to sign an informed consent form and were informed about their right to withdraw for any reason at any stage of the study. Patients’ privacy was completely preserved throughout the study and their data were handled and analyzed in the coded form. The research project was approved by the Ethics Committee of the University of Leipzig. In case of any research-related complication, patients would be fully supported by researchers. Patients aged ≥22 years with sinus rhythm in 12-lead ECG at the time of enrollment were included. The reason for choosing 22 as the minimum age was the information provided on the official website of Apple Watch. Patients with mental or motor impairments that would limit their cooperation in ECG recording and pregnant women were excluded from the study.

### 2.2. Data Collection

Baseline patient characteristics including age, gender, body mass index, and history of diseases and medications were collected. All 12-lead ECGs were recorded by a common ECG device (Edan SE-1200 Express, CAmed Medical Systems GmbH, Köln, Germany) with a paper running speed of 25 mm/s and amplitude of 10 mV/mm over 30 s. The filter parameters of the standard ECG recorders were set to 0.67 Hz cutoff for low-frequency filtering and 150 Hz cutoff for high-frequency filtering. All standard ECGs were immediately evaluated by a cardiologist, and patients with sinus rhythm were considered eligible for obtaining ECG with the smartwatch.

### 2.3. ECG Recording with Smartwatch

**Figure 1:**
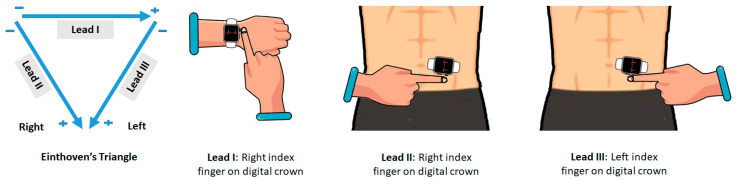
The positioning of the Apple Watch Series 4 for obtaining leads 1, II, and III using the Einthoven triangle. In the Apple Watch, the negative electrode is placed in the digital crown and the positive electrode is on the back crystal of the watch.

For eligible patients, smartwatch ECGs were obtained immediately after the standard ECG without any change in patient condition. The smartwatch used in this study was the Apple Watch Series 4 (watch OS 5.1.2). The smartwatch ECG recording speed was set to a speed of 25 mm/s and an amplitude of 10 mV/mm. The watch is equipped with two electrodes: (1) a negative electrode, which is built into the digital crown; and (2) a positive electrode, which is placed on the back side of the watch. All steps of ECG recording with smartwatch were undertaken by the patients themselves under the supervision of a research assistant. All patients were informed in advance about how to record ECG with the smartwatch. To record lead I, patients were asked to wear the watch on the left wrist and place their right index finger on the digital crown of the watch and wait for 30 s, so that ECG can be recorded (Figure 1). In order to record lead II, patients were asked to place the watch on the lower abdomen and place their right index finger on the digital crown for 30 s. Finally, to record the Lead III, patients were asked to place their left index finger on the digital crown for 30 s without changing the position of the watch on the abdomen These positions were selected based on the principles of the Einthoven triangle (Figure 1). All stages of ECG recording were performed with an Apple Watch and an Apple market version of the ECG app (Apple Inc., Cupertino, CA, USA), while the device was paired to an Apple iPhone 7 via Bluetooth.

### 2.4. Data Transfer

All ECG data recorded by the smartwatch were automatically transferred to the Health app on the paired iPhone. All of these ECGs were also exported as PDF files. These files and the scanned images of the 12-lead ECG were coded and sent to our telemonitoring center for further analysis.

### 2.5. Data Processing

**Figure 2:**
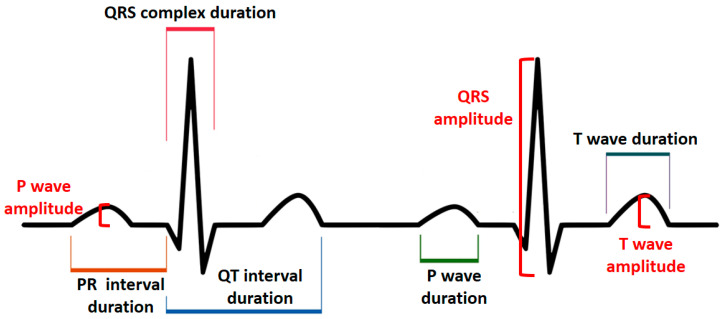
Principles of electrocardiogram assessment.

The lead I–III tracings from the standard ECG and their corresponding tracings from the smartwatch were analyzed by a cardiologist. First, the overall quality of each ECG tracing was assessed. Tracings were considered acceptable for interpretation if baseline artifacts were absent for at least 80% of each tracing. Baseline artifacts were defined as ECG segments in which P waves, QRS, or both complexes could not be identified. To determine the heart rate, the number of QRS complexes, including fractional QRS complexes, in a 6-s interval from lead I was counted, and then it was multiplied by 10 to calculate the heart rate in one minute. The heart rate was calculated separately for each of the ECG recording methods. The heart rate reported by the Apple Watch app was also separately recorded. Tracings were assessed in terms of the following variables based on the previously described methods [22,23] and according to measurement instructions given in Figure 2: (1) duration of P wave, PR interval, QRS complex, QT interval, T wave; (2) amplitude of P wave, QRS complex, T wave; (3) polarity of P wave, QRS complex, and T wave. Durations were recorded in milliseconds (ms), amplitudes were recorded in millivolts (mv), and polarities were recorded as positive and negative. Three different heartbeats were randomly selected within the same tracing, and the segments’ characteristics of each of these heartbeats were determined. The average of these three measurements was then considered as the final value. All measurements were performed using a professional ECG ruler on the printed version of ECGs. Since both methods had an electrical event recording speed of 25 mm/s and amplitude of 10 mV/mm, each millimeter (small box) was equivalent to 0.04 s on the horizontal axis and 0.1 millivolts on the vertical axis. 

Although standard ECGs and smartwatch ECGs could be differentiated from their appearance, at no stage did the reader know which standard ECG corresponds to which smartwatch ECG. 

To prevent bias in the reporting of the results, all ECGs were coded and categorized by another person, and all measurements were blinded. To reduce measurement error, all readings for both standard ECGs and smartwatch ECGs were done by a single experienced cardiologist. Before the actual readings, the accuracy of the cardiologist in reading ECGs was evaluated and verified by an experienced electrophysiologist using 20 random test records.

### 2.6. Statistical Analysis

Tracings of standard lead I, II, and III were used as the reference. Data normality was studied using the one-sample Kolmogorov-Smirnov test. Quantitative data including duration and amplitude of ECG waves were reported in the form of mean ± standard deviation (SD). Qualitative variables including gender, disease and medication history, ECG tracing quality, and wave polarity were expressed as numbers (%). Agreement between the two methods of measurements was calculated using Bland-Altman analyses. Spearman correlation test was used to find correlations between quantitative results of the ECG recording methods, while Fisher’s exact and Chi-square tests were used for qualitative data. The correlation coefficient (r) values were interpreted as follows: values ≤ 0.20 as no agreement, 0.21–0.40 as weak, 0.41–0.60 as moderate, 0.61–0.80 as strong, 0.81–1.00 as very strong correlation. The analytical software used in this study was SPSS version 17 (SSPS Inc., Chicago, IL, USA) and GraphPad Prism version 8.4.2 (San Diego, CA, USA). In all tests, *p*-values < 0.05 were considered statistically significant. 

## 3. Results

### 3.1. Baseline Characteristics

The study was performed on 100 patients (62 male/38 female) (Table 1). A total of 300 smartwatch ECGs were compared with their counterparts from the standard ECGs. For all patients, lead I-III ECGs were recorded without any problem by the smartwatch. All smartwatch and standard ECG tracings were of good quality and had no artifact.

### 3.2. Heart Rate

The mean heart rate calculated from standard ECGs was 77.04 ± 14.41 beats per minute (bpm), for smartwatch ECGs it was 77.52 ± 15.09 bpm, and the App reported this rate as 78.14 ± 15.21 bpm. There was no significant difference between the average heart rate obtained from the standard ECG and those from the smartwatch (p = 0.446, paired student *t*-test), or the paired mobile application (p = 0.079, paired student *t*-test). A very strong correlation was found between the heart rate from standard ECGs and those from smartwatch ECGs and application report (p = 0.000, r = 0.84 and p = 0.000, r = 0.85, respectively).

### 3.3. Duration, Amplitude, and Polarity

**Figure 3:**
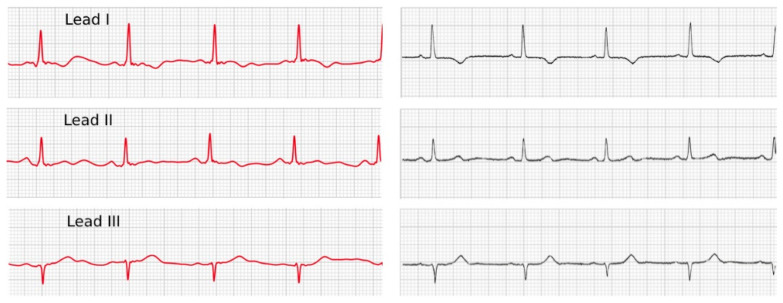
Comparison of a 3-lead ECG recorded by an Apple Watch 4 (**left side**) and a standard ECG device (**right side**).

Standard and smartwatch ECG characteristics are summarized in Table 2 and Table 3. A very strong correlation (r > 0.80) was observed between the duration of waves obtained from the two methods in all three different leads; however, these correlations were reported strong for P-wave amplitude in lead I (r = 0.78) for T wave amplitude in lead III (r = 0.82) (Table 2). Examining the polarity of the evaluated segments revealed a strong association and concordance between the results of smartwatch ECG and standard ECG in all three leads (Table 3, Figure 3). The bias and the range of agreement between the two measurements are summarized in Table 2.

## 4. Discussion

This study aimed to assess the feasibility and reliability of 3-lead ECG recordings by smartwatch devices as compared to a standard ECG. Our results showed that the tested smartwatch could obtain classical Einthoven lead I-III ECGs similar to standard ECGs. Although this is not the first report on the ability of handheld ECG recorders to record leads other than lead I, the following features distinguish it from other studies:

1. This was the first study to quantitatively assess the similarity of lead I, II, and III tracings recorded by the smartwatch with those obtained from the standard ECG, and the first to show not only strong correlation in every three leads but also no clinical difference between the values obtained for each segment. One study has demonstrated the accuracy of the cabled device AliveCor^®^ (AliveCor, CA, USA) in recording different ECG leads in six patients, but in this study, the similarity of smartwatch ECG to standard ECG was assessed qualitatively based on the appearance of ECG recordings [24]. In another study, the handheld device Beurer^®^ ME80 (Beurer GmbH, Ulm, Germany) was found capable of recording different ECG leads, but the analyses of this study were also based on inter-observer agreement and emphasized apparent similarities, rather than making quantitative comparisons between recorded segments [25].

2. Our study showed that using the left lower abdomen instead of the left lower limb can contribute to obtaining high-quality signals, which is consistent with the principles of Einthoven leads II and III in the standard ECG [26]. Although we are not the first group who reported the utility and description of this technique, our study has confirmed its validity in a relatively large sample. Our previous understanding regarding the smartwatch ECGs were limited to the findings of several case reports and case series [7,27,28,29,30], and three studies on around 50–100 patients [13,14,15].

The current method that we used in this study to record a 3-lead ECG was previously described by Samol et al. [14] and Cobos-Gil [12]. In a study by Samol et al., 50 healthy individuals were enrolled and the similarity of 3-lead ECG recordings by an Apple Watch Series 4, encompassing Einthoven’s leads I to III, was compared to the same leads obtained by a standard ECG set [14]. This group of researchers further assessed their method of obtaining ECG with a smartwatch in a larger study of 100 patients [13] and another study of 50 healthy individuals [15]. Both of these two studies highlighted that the quality of 3-lead ECGs obtained by an Apple Watch Series 4 was adequate in more than ≈90% of the recordings, and Apple Watch ECGs were quite similar to standard ECG recordings. However, the main difference between these two studies and what we did in our research was the method of comparison between the two methods of ECG recording. In our study, a quantitative approach was used; however, in the aforementioned studies, a qualitative approach was taken, where cardiologists were asked to assign the Apple Watch ECGs to the standard ECGs based on the morphology and the amplitude of recorded signals.

3. The current attempt is one of the first studies compared to the smartwatch ECG with the standard ECG in a real-world setting. Although the ability of smartwatches to record ECG was confirmed by FDA in 2018, to the best of our knowledge, no other real-world study has verified the quality of ECG signals recorded by smartwatches with a quantitative approach. The importance of this study also stems from the fact that every year hundreds of thousands of smartwatches are sold across the world, which reflects the higher access of people to this device than to its competitors.

As an interesting finding, the mean age of our included patients was 63.6 ± 14.0 years, which reflects an older age in comparison to other studies [14,15]. This is important because one might think that it would be very difficult for the elderly to record ECG with a smartwatch, but our experience showed that the elderly without motor or mental problems could undertake this task confidently after a very short training. However, it should be noted that our subjects were only asked to record the ECGs with smartwatch, not to email the resulting files or export them to another device, which could be bigger challenging tasks for the elderly.

**Figure 4:**
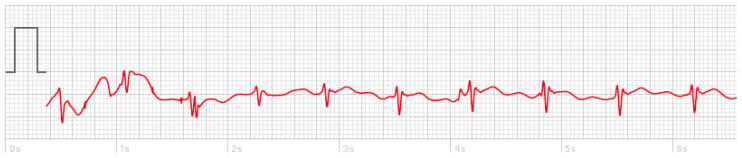
A sample of temporary artifact (around one second) due to instability of the Apple Watch 4 on a patient’s wrist.

Another interesting point was the exceptionally high quality of smartwatch ECG. Specifically, none of the ECG tracings obtained from the smartwatch contained significant baseline artifacts. However, instability of the smartwatch on the patient’s wrist or abdomen could create artifacts for a few seconds, which in most cases disappeared after stabilizing the smartwatch (Figure 4). However, it should be noted that the quality of ECG signals can be affected not only by the settings of the ECG recorder, but also by other factors including skin preparation prior to the placement of electrodes. The present study was conducted without any specific skin preparation protocol and without considering skin greasiness and hair growth, which make the measurement process more akin to the real-world experience. Further, the manufacturer of the Apple Watch has not specified any skin condition requirements for ECG recording.

The findings of this study suggest that patients will be able to record their own 3-lead ECGs without needing to attach additional cables to their bodies by using a smartwatch in any situation. We believe that this study will not only promote the use of smartwatch ECG in the diagnosis of arrhythmias, but may also contribute to early detection of inferior myocardial infarction in patients with manifestations of acute coronary syndrome. A very interesting example of the use of the Apple Watch in the setting of acute coronary syndrome was published months ago [29]. In this case report, physicians decided to omit further diagnostic procedures in the chest pain unit and to perform the angiography in an 80-year patient presented with angina symptoms based on her smartwatch recordings showing ST-segment depression, although the initial 12-channel ECG and the high-sensitive troponin I test result were not suggestive of myocardial ischemia. During the angiography, a significant stenosis in the left main stem and a lesion in the left anterior descending/diagonal bifurcation were diagnosed [29]. In another example, the feasibility of Apple Watch ECG recordings for the detection of cardiac ischemia in two patients with ST-elevation myocardial infarction was approved [7]. In this study, a 3-lead ECG was obtained by using a similar method, which is used in our current study. In both of the patients, the real-time, 3-lead Apple Watch ECG recordings were compatible with the standard ECG waveforms, indicating an inferior myocardial infarction [7]. It has been shown that the late arrival of patients at diagnostic centers is one of the most common causes of late diagnosis and treatment of ST-elevation myocardial infarction [31]. Indeed, a rapid ECG, followed by timely transfer to specialized centers can, in many cases, lead to the rapid diagnosis of ST-elevation myocardial infarction and reduce the door-to-needle or door-to-balloon time [32]. Hence, improving access to ECG can be an effective measure for early diagnosis and treatment of this condition. Therefore, smartwatch ECG recordings might be the solution.

While the arrhythmia symptoms of patients are often absent when they visit physicians, smartwatches with the capability of ECG recording can be an effective strategy to catch and record more accurate evidence during the emergence of symptoms at any location [4,5,9,10]. Recently, validity of the use of the Apple Watch for screening and monitoring of QT prolongation has been also reported [33]. In this study, the smartwatch was placed on the left ankle or left lateral chest to produced leads II and V6. Similar to our findings, no clinically significant differences in between what were recorded by the Apple Watch and the standard ECG. However, the findings of this study were limited to the QTc-interval and T-wave amplitude in three leads, including lead I, II and V6. 

The expansion of the function of smartwatches from single-lead to 3-lead ECG recording can be especially helpful in cases where patients do not have immediate access to health-care facilities. In these situations, patients can take a 3-lead ECG and share it with a health-care provider, in order to receive further instructions. However, it is also important to educated patients about the fact that the current smartwatches cannot replace standard ECG or provide an accurate interpretation of ECG records like a physician. We believe that what is happening to smartwatch ECG is somewhat similar to what has already happened to other personal health monitoring devices, such as home glucose and blood pressure monitors, as there were some concerns about their utility and reliability when they were introduced to the market, but, gradually, it became clear how much these tools can help reduce medical costs and improve the outcomes of medical interventions [2]. Therefore, a more sensible reaction to the advent of smart devices with medical functions is to contribute to improving their utility and performance, instead of resisting technological progress.

### Limitations

(1) This study was performed only on individuals with sinus rhythm to make sure that the results obtained from the two methods are comparable. However, this restriction of the sample to subjects with sinus rhythm highlights the need for additional study to assess the accuracy of smartwatch EGs for detection of different types of arrhythmias or ECG changes. (2) The process of recording ECG with the smartwatch was supervised by the researchers. Therefore, ECG tracings obtained from smartwatch without such supervision may not have the same degree of feasibility or quality. (3) One of the factors that may affect the morphology of ECG signals is the frequency filtering parameter of the recorder system [34]. Although our study showed a strong correlation in terms of morphology between standard ECGs and those of the smartwatch, their slight differences can be due to possible differences in these parameters; an issue that could not be further investigated, because the filtering parameters of the Apple Watch were not available to us. (4) The results of this study are limited to one particular model of smartwatch, that is, the Apple Watch Series 4, and may not be generalized to other devices.

## 5. Conclusions

The results of this study suggested that the examined smartwatch (Apple Watch Series 4) could obtain 3-lead ECGs, including Einthoven leads I, II, and III by placing the watch on the described positions. Quantitative analysis showed a strong correlation between the tracings obtained from smartwatch ECG and standard ECG for all of the examined segments in all three leads. Therefore, this approach can be recommended as a new strategy for faster detection of cardiac disorders that can be diagnosed from lead I-III ECG, especially when there is no immediate access to standard ECG facilities.

## Figures and Tables

**Table 1:** Patients baseline characteristics.

Variables, Units	n (%)
Age (year), mean ± SD	63.66 ± 14.00
Gender (male/female), n	62/38
Body mass index (kg/m^2^), mean ± SD	28.44 ± 4.77
Ischemic heart disease	41 (41)
Hypertension	88 (88)
Diabetes	31 (31)
Prior stroke	10 (10)
Renal failure	31 (31)
Chronic obstructive pulmonary disease	10 (10)
Beta-blocker	82 (82)
Digoxin	3 (3)
Amiodarone	16 (16)
Diuretic	55 (55)
Angiotensin receptor blocker (ARB)	31 (31)
Angiotensin-converting enzyme (ACE)-inhibitor	39 (39)
Antiplatelet drug	33 (33)
Anticoagulant	62 (62)

**Table 2:** Electrocardiogram characteristics (duration and amplitude).

Variables, Units		Lead	Standard ECG	Smartwatch ECG	Correlation Coefficient	*p*-Value	Bias * (95% LoA)
**Duration (millisecond), mean ± SD**	**P Wave**	I	76.79 ± 11.69	76.54 ± 11.58	0.94	0.001	0.25 (−7.44 to +7.94)
		II	77.27 ± 13.15	77.89 ± 13.15	0.94	0.001	−0.62 (−9.82 to +8.58)
		III	77.39 ± 14.66	78.64 ± 13.91	0.96	0.001	−0.01 (−7.28 to +7.26)
	**PR Interval**	I	172.21 ± 24.55	171.36 ± 24.69	0.98	0.001	0.85 (−8.01 to +9.71)
		II	173.73 ± 29.99	172.69 ± 29.17	0.97	0.001	1.04 (−11.55 to +13.63)
		III	172.07 ± 28.26	172.65 ± 27.62	0.96	0.001	−0.68 (−16.32 to +14.95)
	**QRS Complex**	I	87.44 ± 11.70	87.67 ± 11.18	0.94	0.001	−0.23 (−7.86 to +7.39)
		II	88.84 ± 10.73	89.01 ± 10.46	0.93	0.001	−0.17 (−8.18 to +7.84)
		III	87.08 ± 14.54	87.23 ± 14.00	0.95	0.001	−0.14 (−9.30 to +9.01)
	**QT Interval**	I	369.66 ± 44.26	369.93 ± 44.36	0.99	0.001	0.04 (−11.05 to +11.13)
		II	368.42 ± 42.19	367.44 ± 40.67	0.98	0.001	0.09 (−9.90 to +11.87)
		III	369.57 ± 42.36	369.22 ± 42.18	0.99	0.001	0.35 (−9.98 to +10.96)
	**T Wave**	I	132.41 ± 24.72	133.10 ± 24.81	0.97	0.001	−0.32 (−12.53 to +11.88)
		II	132.81 ± 24.43	133.13 ± 23.93	0.97	0.001	−0.32 (−12.53 to +11.88)
		III	133.76 ± 24.15	133.82 ± 23.17	0.98	0.001	−0.06 (−8.34 to +8.21)
**Amplitude (millivolts), mean ± SD**	**P Wave**	I	0.20 ± 0.06	0.22 ± 0.07	0.78	0.001	−0.01 (−0.10 to +0.07)
		II	0.23 ± 0.09	0.24 ± 0.95	0.89	0.001	−0.01 (-0.09 to +0.08)
		III	0.21 ± 0.08	0.21 ± 0.08	0.83	0.001	−0.01 (−0.09 to +0.09)
	**QRS Complex**	I	1.79 ± 0.68	1.84 ± 0.70	0.96	0.001	−0.04 (−0.38 to +0.28)
		II	1.97 ± 0.84	1.94 ± 0.78	0.89	0.001	0.02 (−0.73 to +0.78)
		III	1.94 ± 0.64	1.97 ± 0.65	0.90	0.001	−0.02 (−0.60 to +0.55)
	**T Wave**	I	0.29 ± 0.15	0.32 ± 0.15	0.89	0.001	−0.02 (−0.16 to +0.11)
		II	0.32 ± 0.17	0.32 ± 0.15	0.82	0.001	0.00 (−0.19 to +0.20)
		III	0.28 ± 0.16	0.29 ± 0.18	0.72	0.001	−0.01 (−0.27 to +0.23)

* Bias was calculated by Bland-Altman analysis; ECG: electrocardiogram; LoA: limits of agreement.

**Table 3:** Electrocardiogram characteristics (polarity).

Variables, Units		Lead	Standard ECG	Smartwatch ECG	Concordance (%)	*p*-Value
**Polarity (Positive/Negative), n**	**P Wave**	I	100/0	100/0	100%	-
		II	99/1	99/1	100%	0.001
		III	93/7	94/6	99%	0.001
	**QRS Complex**	I	94/6	94/6	100%	0.001
		II	88/12	87/13	99%	0.001
		III	54/46	53/47	99%	0.001
	**T Wave**	I	82/18	81/19	99%	0.001
		II	83/17	84/16	99%	0.001
		III	70/30	72/28	98%	0.001
